# Transactions of the Chicago Society of Physicians and Surgeons

**Published:** 1874-06-15

**Authors:** Ralph E. Starkweather


### TRANSACTIONS OF THE CHICAGO SOCIETY OF PHYSICIANS AND SURGEONS.

### REGULAR MEETING, MAY 25TH, 1874.

*Reported by Ralph E. Starkweather, M.D.*

THE Society met, as usual, at the Grand Pacific hotel. Dr. Jno. Bartlett, President, in the chair. On motion of Dr. Owens, the minutes of the previous meeting were approved.

Dr. Trimble was prepared to submit only a partial report from the Board of Censors, and requested that hereafter the members of the Society would comply more strictly with the third article of the Constitution, in reference to giving full information respecting the candidates proposed for admission into the Society.

Doctors J. W. Freer, W. A. Harvey and H. Hooper were unanimously elected members. The names of Doctors H. M. Bannister, H. W. Jones, M. O. Heydock, and H. W. Boyd were proposed, and referred to the Censors.

The customary order of proceedings was now, on motion, suspended, by the presentation and discussion of a series of resolutions and amendments, which will await final action at subsequent meetings.

Dr. Trimble proposed, as an addition to the first article of the Constitution, to insert, "are now or have hitherto been regular practitioners in the city of Chicago, and are not now engaged in other business." This was farther amended by Dr. Owens, seconded by Dr. Hyde, "that no applicants be considered eligible as members of this Society, who have not been engaged in practice three years."

Dr. F. H. Davis thought that the period of three years was rather too long, but would favor an interval of one year, after graduation. It is really the young men who do much of original investigation and study—before they become much engaged in business; and it would discourage such men, were they kept out of societies, unable to present the results of their studies.

Dr. Owens—The limit of three years is approved by many useful members of this Society, not here present this evening.

Dr. Wood—Would like to see the period made three, or even seven, years, as it would elevate the standard of the Society, and make it compare favorably with those of other countries and cities.

Drs. W. C. Lyman and Blake thought the clause relative to following any other business, ought to be excised.

After remarks by Doctors Henrotin and Jackson, Dr. Emmons suggested that the rules of the Association, and the powers of the Censors, covered the subject already.

Dr. Hyde replied, that the Code of Ethics of the American Medical Association does not prescribe who shall be its members—whether a druggist or physician; it simply directs physicians to discourage the selling by druggists of nostrums—patent medicines, and the like.

Dr. Merriman opposed frequent changes in the Constitution as unnecessary. The Society could supplement the action of its Board of Censors, when voting upon membership.

Dr. Hyde objected to the annual overflow of graduates from the colleges pouring into the societies. He would favor the period of two years, as a compromise.

Dr. Blake—It seems to me that it ought to be well understood, that this Society does not admit a person who is not a practicing physician, or who may be a druggist. It is unfair to leave such responsibility to the Censors; they deserve to have our support.

Dr. Trimble said the amendment he offered had no intention of interfering with the private business of the members—except so far as the subject involved the drug business.

Dr. Jackson suggested adding to article I, "who possess a good, moral, and professional reputation; who shall have been practitioners of medicine for two years, and who shall not be interested in the sale of drugs, medicines or surgical appliances."

On motion, the following resolution was adopted:

WHEREAS, In the judgment of this Society, it is unbecoming for a member of the medical profession to engage in the business of a druggist or apothecary, and,

WHEREAS, It is reported that certain members of this Society are about to, or have already thought it proper to, assume a financial interest in the prosecution of the general business of the sale of drugs and medicines; therefore,

*Resolved*, That the Censors of the Society are hereby requested to ascertain if such reports implicate any of its members; and if yea, to enquire of such members, respecting the fact of such traffic or business. And, if these allegations be true, they shall be informed by the Censors that they will be allowed to resign honorably to themselves, during the period of one month from the date of such inquiries; and,

*Resolved*, That in case any member who has been found to be engaged, or interested financially, in the business described, does not confess to the same, or refuse to resign the membership in one month, the Censors shall forthwith proceed to bring such member to trial, in the form and manner prescribed by the Constitution and By-laws of this Society.

Dr. Hyde, in behalf of Dr. F. H. Davis, presented the report of the Banquet Committee, with vouchers, which was accepted, and a vote of thanks to the committee carried.

It was voted, that delegates from this Society, to the State and American Medical Societies, be elected at the annual meeting.

The President announced the following appointments to the Sections:

1. On the Practice of Medicine, to report July 13, 1874—Dr. Walter Hay, Chairman; Drs. J. H. Hollister, W. C. Lyman, W. M. Boyd.

2. On Materia Medica and Therapeutics—Dr. D. B. Trimble, Chairman; Drs. J. H. Etheridge, F. H. Davis, H. P. Merriman.

3. Section on Surgery—Dr. E. Andrews, Chairman; Drs. E. Powell, H. McKennan, C. T. Parkes.

4. General Pathology, to report January 11, 1875—Dr. I. N. Danforth, Chairman; Drs. Lester Curtis, C. P. Simon, F. Henrotin, Jr.

5. Obstetrics and Diseases of Women—Dr. DeLaskie Miller, Chairman; Drs. A. Reeves Jackson, A. P. Peck, M. W. Wood.

On motion, the Society adjourned.

---

MEETING JUNE 8, 1874.

The following gentlemen were elected to membership: Doctors M. O. Heydock, H. M. Bannister, H. W. Jones and D. A. K. Steele. The names of Drs. H. A. Johnson and H. K. Newton were proposed and referred.

Dr. Hyde read a paper, "Notes on the Microscopical Appearances of the Brains of the Insane," prepared by Dr. Walter Kempster, of the Northern Asylum for the Insane, at Oshkosh, Wisconsin, formerly of the New York State Lunatic Asylum, at Utica. He had made microscopical examinations in forty-nine cases. Numerous slides were exhibited of sections, made mostly through the third left anterior cerebral convolution, illustrating the lesions of acute mania; the large sclerous patches in chronic mania; the dementia of syphilitic paralysis; one section through the olivary body, and one through the pons varolii—each illustrative of acute mania.

Numerous micro-photographs were likewise shown, illustrating the lesions of cerebro-spinal meningitis; of numerous colloid masses in the medulla oblongata, and large degenerated masses with dense fibrous investing membrane in the spinal cord, opposite second cervical vertebra—each illustrative of acute mania. Also, a section through the olivary bodies, in a case of puerperal mania, showing fibres and connective tissue in degenerated masses.

After acknowledging the great abilities and researches of Lockhart, Clarke, Virchow, Meynert, Shultze, Deiters, and others, in the study of the nervous system, Dr. Kempster remarks that, so far as he is aware, none of them have directed especial attention to the abnormalities found in the brains of those who die while insane.

Reference was made to an article in the *Edinburgh Medical Journal* for September, 1868, by Dr. J. B. Tuke, as being the only exception which Dr. Kempster was able to find.

The student is met with the stereotyped phrase that there are no discernable lesions peculiar to insanity. For a number of years Dr. Kempster has been making systematic microscopical study of the brain, and has examined the lesions of all forms of insanity, from acute mania to dementia, including puerperal and epileptic insanity.

In each and all forms he has found a marked lesion—so that certain lesions may be grouped together as common to certain forms of insanity, and to which lesions any particular type of insanity is palpably due. There is a wide difference between the lesions of acute and chronic mania.

I. In certain forms of insanity, and notably in dementia, the finer capillaries show marked indications of disease, the peri-vascular sheath surrounding the vessel is distended, so much so, that sometimes the vessel itself appears to lay in a tunnel, its calibre being much less than the sheath, doubtless due to repeated capillary congestions of the vessels often diseased—irregular in calibre, suggesting the idea of aneurismal dilatations, but entirely distinct from the miliary aneurisms so ably described by Charcot.

II. Next, there is a degeneration, best studied in cases of dementia of syphilitic origin, and in the medulla oblongata, in the wall of the capillary, presenting dark red patches at various points outside its walls, which gradually thicken, and appear to be due to a fatty metamorphosis or atheroma. The description by Meynert, though accurate, is by no means so complete as could be desired.

III. In 1871, while examining a section taken from the grey and white matter of the third left anterior convolution, there was a peculiar appearance of the tissue. Situated in the white substance, but very closely to the grey matter, there were a number of small *white spots*, some round, some ovoid, clearly defined, in sharp contrast with the nerve tissue, varying in size, from 1-50 to 1-200 of an inch in diameter—these appeared to be of a granular consistence, and much more dense in structure than the surrounding brain substance; each disconnected from the other, and normal white matter intervening. They did not absorb carmine, and were not connected with the capillaries. On the surface of some of the spots are fibres of connective tissue and crystals of margarine. To determine the true character of these spots and the degeneration, certain very elaborate and extensive micro-chemical manipulations were made, not here necessary to be stated. On allowing a section to dry, either with or without the nitric acid treatment, these spots appear to project above the surface of the section. By teasing, they may with difficulty be removed. None of these spots have been observed in the grey matter. They are most numerous in the medulla oblongata, and may be found in the white matter of the spinal cord.

IV. There is another form of degeneracy, one which was found in cases of acute mania. The spots are less in size; are far more numerous than in the other variety (3); resist carmine staining; do not possess the granular characteristic; there are no spindle-shaped fibres of connective tissues about them; they behave very differently under the micro-chemical tests applied to the other variety of spots. The points of resemblance are mainly in color and apparent density. Neither of them have any investing membrane.

V. A fifth variety, as large in size as the third, possesses a dense investing membrane, which resists carmine staining and is less granular than the third and fourth. It exists in the same brain with the fourth variety. These spots or masses of the fifth variety are called "colloid," because of their resemblance to such growth, and are found in the medulla oblongata and pons varolii. The last three varieties of degenerated masses, or spots, have one feature in common—a well-defined edge, a clean-cut margin, easily made out.

VI. A sixth variety, common in cases of dementia, and where the atheromatous capillary is found, is one in which the mass passes insensibly into the surrounding normal tissues. This form is larger and less distinct than the others. It more nearly resembles normal brain tissues. Sometimes these masses are lobulated. They are granular and dense, less numerous than in the other varieties, and do not appear in clusters. They appear to destroy or transform the tissues, and if surrounding a capillary, destroys its walls. A point of resemblance in common with the third variety is, that connective tissue fibre appears in both.

The condition of the cellular structures of the brain, of the nerve-fibres and so-called lymph-spaces, are all fields rich in results not here spoken of.

A resolution of thanks to Dr. Kempster, and his election as honorary member of the Society, were unanimously carried. The amendment to Article One of the Constitution, proposed by Dr. Jackson at the last meeting, was taken up and accepted.

---

ICED WATER INJECTIONS IN ACUTE DYSENTERY.—In the *Berliner Klin Wochenschrift*, December, 1873, Dr. Wenzel has a communication regarding the favorable results with which he has employed clysters of iced water in relieving the most severe hemorrhage and tenesmus in a great number of cases of *acute* dysentery. The enemata may be rendered still more efficacious by the addition of finely *shaved* or pounded ice.

AT the fifth annual meeting of the French Association for Opposing the Abuse of Alcohol and Tobacco, the President, Jules Guerin, called especial attention to the falling off in the use of both tobacco and spirits, as evinced by the serious deficit in that department. Additional taxes have recently been imposed upon both these articles, which, in his opinion, would cause a still further decrease of their use.

